# The Functional Role of microRNAs in the Pathogenesis of Tauopathy

**DOI:** 10.3390/cells9102262

**Published:** 2020-10-09

**Authors:** Domenico Praticò

**Affiliations:** Alzheimer’s Center at Temple, Lewis Katz School of Medicine, Temple University, Philadelphia, PA 19140, USA; praticod@temple.edu

**Keywords:** microRNA, Alzheimer’s disease, progressive supranuclear palsy, corticobasal degeneration, Pick’s disease, tau protein, tau phosphorylation, gene regulation

## Abstract

Tauopathies are neurodegenerative disorders which include Alzheimer’s disease, Pick’s disease, corticobasal degeneration, and progressive supranuclear palsy among others. Pathologically, they are characterized by the accumulation of highly phosphorylated and aggregated tau protein in different brain regions. Currently, the mechanisms responsible for their pathogenesis are not known, and for this reason, there is no cure. MicroRNAs (miRNAs) are abundantly present in the central nervous system where they act as master regulators of pathways considered important for tau post-translational modifications, metabolism, and clearance. Although in recent years, several miRNAs have been reported to be altered in tauopathy, we still do not know whether these changes contribute to the onset and progression of the disorder, or are secondary events following the development of tau neuropathology. Additionally, since miRNAs are relatively stable in biological fluids and their measurement is easy and non-invasive, these small molecules hold the potential to function as biomarkers for tauopathy. Herein, we showcase recent findings on the biological link between miRNAs and the pathogenesis of tauopathy, and present emerging evidence supporting their role as biomarkers and targets for novel therapies against them.

## 1. Tauopathies

Tauopathies are heterogeneous neurodegenerative disorders whose main pathologic feature is characterized by the accumulation of phosphorylated and misfolded tau protein in the brain parenchyma [1]. Clinically, they manifest with progressive memory and learning deficits and in some cases also motor function impairments. In general, these symptoms manifest when the subject is about 55–60 years old, and deteriorate within an average of 10 years from the onset [2]. The most common forms of tauopathy are: Alzheimer’s disease (AD), Down syndrome, progressive supranuclear palsy (PSP), corticobasal degeneration (CBD), Pick’s disease, and frontotemporal dementias with Parkinsonism linked to chromosome 17 (FTDP-17). They are mainly sporadic diseases and while age represents the strongest risk factor, other risk factors are considered: gender, traumatic brain injury, cardiovascular diseases, and hypertension [3,4,5]. Currently, the etiology of these disorders as well as the molecular events responsible for tau hype-phosphorylation, tau aggregation, and ultimately neurodegeneration are still unknown, and for this reason, there are no effective therapies against them. A timely and accurate diagnosis for these conditions is generally difficult because of the overlapping clinical phenotypic manifestations among them particularly at the early stages, and the unavailability of biomarkers with good specificity and sensitivity. Besides family history and cognitive evaluation, neuroimaging techniques such as positron emission tomography (PET) and magnetic resonance imaging (MRI) looking at tau deposition and distribution today represent the best available tools to make a diagnosis of and to discriminate among tauopathies.

Based on the pathology, in general, these diseases are classified in two groups: primary (PSP, Pick disease and CBD, FTDP-17), when tau is the main lesion, or secondary (AD, Down syndrome), when tau is associated with other pathologies (i.e., Aβ) (Table 1). In recent years, several studies have shown that, in Parkinson’s disease (PD), besides the classical α-synuclein pathology, there is also clear tau pathology accumulation [6,7]. In this sense, although not a classical tauopathy, PD could be also classified as a secondary one. Additionally, these diseases can be sub-divided taking into consideration the ratio of the different tau repeats (R), 3R/4R. In fact, alternative splicing of exon 10 of the tau gene results in three or four repeat domains (3R and 4R, respectively) [8]. For instance, AD and Down syndrome are considered mixed 3R and 4R tauopathies. A prevalence of the 3R isoform of tau is typical of Pick’s disease, whereas 4R repeats are common in CBD and PSP (Table 1). Another aspect of tau pathology that shows some disease specificity is the structure and localization within the cellular compartments. Thus, paired helical filaments and straight filaments are typically found in AD and they are present inside neurons as neurofibrillary tangles [9] (Table 1). On the other hand, abundant rounded cytoplasmic neuronal inclusions, composed of tau straight filaments, called Pick’s bodies, and globular oligodendroglial inclusions are found in Pick’s disease. Finally, tau can also accumulate in neurons and glia cells, as tufted astrocytes and astrocytic plaque, in both PSP and CBD (Table 1) [10].

Based on post-mortem assessment of tau neuropathology, these disorders also have a specific pattern of tau spreading. In PSP tau pathology is prominent in the brainstem, sub-thalamic nucleus and dentate nucleus of cerebellum. In CBD, tau inclusions are observed through the basal ganglia and brainstem regions, while in Pick’s disease through the limbic system and neocortical areas [11]. On the other hand, in AD the neuroanatomical pattern of tau spreading is well characterized across the six Braak stages. In stage I, lesions develop in the trans-entorhinal region to extend only during stage II into the entorhinal cortex and hippocampal structures. During stages III–IV tau pathology is more severe reaching the adjoining temporal neocortex of the occipito-temporal and lingual gyri in the limbic allocortex and adjoining neocortex. Finally, in stages V–VI, tau lesions appear also in secondary and primary neocortical areas, in the occipital lobe, and extend into the striate area [12].

Although tauopathies are mainly sporadic diseases, more than 40 mutations of the tau gene have been identified, including missense, intronic, silent mutations and single-codon deletions [13]. Some of them influence tau alternative splicing of exon 10, and thus the abundance of different repeats and consequently their ratio (Table 1), while others impair protein–protein interaction, tau binding to microtubules, and can promote the formation of filaments [13].

## 2. miRNAs Biogenesis and Functions

MicroRNAs (miRNAs) are endogenous RNA molecules of 21–23 nucleotides that regulate gene expression at the post-translation level [14]. They belong to a relatively new class of RNAs which also includes: small-interfering RNAs, piwi-associated small RNAs, long-non-coding RNAs and circular RNAs. They are not translated into proteins and for this reason they are also referred to as non-coding RNAs. MiRNAs are transcribed by RNA polymerase II as single strand RNA primary transcripts (pri-miRNAs). The pri-miRNAs are further processed by the Drosha (RNase III enzyme)/DiGeorge Syndrome Critical Region 8 (DGCR8) complex in the nucleus leading to the generation of a hairpin-shaped precursor miRNA called pre-miRNA. Pre-miRNAs are then actively exported to the cytoplasm, where Dicer, another RNase III in association with the RNA binding protein TRBP generates the double-stranded 22nt long mature form of miRNAs [14,15,16]. At this point, one strand of the miRNA will be recognized by the RNA-silencing complex (RISC) and will guide RISC to the 3′UTR of target mRNAs leading to their translational repression or mRNA degradation. As a result, a single miRNA can regulate the translation of a large number of mRNAs, whereas one mRNA can be under the control of different miRNAs [14,15,16].

These non-coding RNAs are abundantly present in the central nervous system where they show a region-dependent expression pattern [17,18]. For instance, miRNA expression profiling analysis showed that a subset of miRNAs is expressed specifically in the hippocampus and in the cortex of adult mice [19]. Moreover, distinct miRNAs expression profiles depend on the neuronal subtypes (glutamatergic vs. GABAergic neurons), and the cellular compartment (distal axons vs. synaptic fraction). Examples of miRNAs enriched in the synapse include miR-200c, miR-339, miR-322, miR-318, miR-29a, miR-7, and miR-137 [20], while miRNAs enriched in distal axons are miR-15bm, miR-16, miR204, and miR-221 [21]. Because of this highly specific expression pattern, miRNAs are thought to be involved in precise neuronal functions. In fact, growing evidence confirms that miRNAs are master regulators of pathways important for brain function like neurogenesis, synaptic function, neuronal survival, and memory [17,18]. Through studies of gain and loss of function, several miRNAs have been identified as important for neurogenesis and axonal development, such as miR-124 and miR-9 [22,23,24,25,26]. Evidence that miRNAs regulate memory and learning comes from neuronal specific inducible deletion of Dicer in mice, which results in changes of behavior and affects the morphology of dendritic spines and levels of synaptic proteins (i.e., BDNF and PSD95) [27,28,29]. Interestingly, recent works have highlighted the more general role that Dicer also plays in neurodegenerative diseases, including PD, as an important modulator of cell survival and neurogenesis [30,31,32].

The miR-132 has been shown to regulate dendritic arborization and synaptogenesis, and affect behavior in mice as well [33,34]. Other miRNAs implicated in regulation of memory are: miR-128b, which influences the formation of fear-extinction memory [35], miR-124, which is involved in sensory motor memory and serotonin-induced synaptic plasticity via CREB signaling [36], and miR-134, which affects long term memory targeting CREB mRNA and causes reduction of BDNF expression [37]. Finally, miRNAs have also been involved in neuroinflammation. For example, miR-146a is a negative regulator of inflammation by inhibiting toll like receptor 4 (TLR4) signaling pathway [38,39]. By contrast, depending on the cellular context miR-155 exerts both pro- and anti- inflammatory properties [39,40].

## 3. miRNA Dysregulation in Tauopathies

In recent years, a large number of studies have shown that compared with controls miRNA levels are altered in several neurodegenerative disorders [41,42]. These findings have attracted the interest of the scientific community and opened up to two main areas of research focused on miRNAs as biomarkers and miRNAs as therapeutic targets. As the most prevalent form of tauopathy, the majority of the published studies so far have analyzed the expression level of different miRNAs in brain, plasma, and cerebrospinal fluid (CSF) of AD patients with a relative scarcity of data for primary tauopathies [43,44].

As mentioned before, currently, there are no reliable biomarkers that can be used to diagnose these diseases. Since miRNAs are present in a stable form in biological fluids such as plasma, serum, and CSF and their measurement is relatively easy and non-invasive, they potentially represent an ideal biomarker candidate for tauopathies. According to the literature, the most promising AD-related miRNA biomarkers include: miR-455-3p, miR-34a-5p, and miR-146a. The miR455-3p is consistently up-regulated in the brain, serum, and plasma of AD patients [45], while miR-34a-5p and miR-146a are up-regulated in the brain but down-regulated in plasma and CSF of AD subjects [46,47]. The expression of miR-132 originally was found to be down-regulated in temporal, parietal, and prefrontal lobes of PSP patients compared to controls [48]. However, this observation was not confirmed in another study looking at PSP patients and controls [49]. Based on another report, CSF levels of miR-106b-5p discriminated between PSP and PD patients with a good clinical accuracy [50]. Finally, miR-9, miR-29b, miR-34a, miR-146a, and miR-125b were assessed in the sera of AD patients in relation to changes in cognition and cerebral cortex integrity [51,52,53]. The authors found that lower serum expression of miR-9, mir-34a, and miR-125b is associated with changes in cognitive performance, cortical thickness, and abnormal cortical glucose metabolism in normal elderly subjects [53].

Taken together, these data suggest that changes in miRNAs can identify different signs of brain aging, even in healthy individuals, and for this reason they have the potential to be used as biomarkers [54,55]. Nevertheless, it is important to note that this area of investigation is still in its infancy and that there are several important challenges ahead. Among them, the most obvious is probably the ability and validity of translating any findings or measures of miRNAs in CSF or blood into a real clinical setting. Another issue that needs to be solved is the assessment of the specificity and sensitivity level that each miRNA found dysregulated achieves in a particular disease state when compared with healthy controls and, most importantly, other neurodegenerative diseases. Moreover, variability and reproducibility issues are often present when comparing different studies. Thus, while a single miRNA is often confirmed and validated by other independent studies, the so called “miRNA signature” (a group of specific miRNAs) of a particular disorder is not.

## 4. miRNAs as Therapeutic Strategy in Tauopathy

Given their role as master regulators of the expression of many genes important for neurodegeneration, the manipulation of miRNA levels is obviously a promising therapeutic strategy for tauopathies. In fact, depending on the direction of the miRNA dysregulation that is found in the particular disease, mimics or antagonists could be employed to restore specific miRNAs and indirectly their targets to normal levels. Several in vitro studies and abundant preclinical data in different transgenic mouse models of these diseases provide a strong rationale for this possibility.

One of the first papers testing the hypothesis that miRNA loss of function could result in the disruption of the tau metabolic pathway used a conditional Dicer knockout to generate significant changes in miRNA levels in the forebrain of mice [56]. Interestingly, these mice displayed significant decrease in the size of both cortex and hippocampus but abundant neuronal loss only in the hippocampal CA3/4 regions. Moreover, they also had a site-specific and age-dependent hyper-phosphorylation of tau in the cortex and hippocampus, which was associated with up-regulation of mitogen-activated protein kinase 3 (MAPK3/ERK1) [56]. Another study in *Drosophila* reported that loss of miRNA results in neurodegeneration through induction of tau [57].

**Figure 1:**
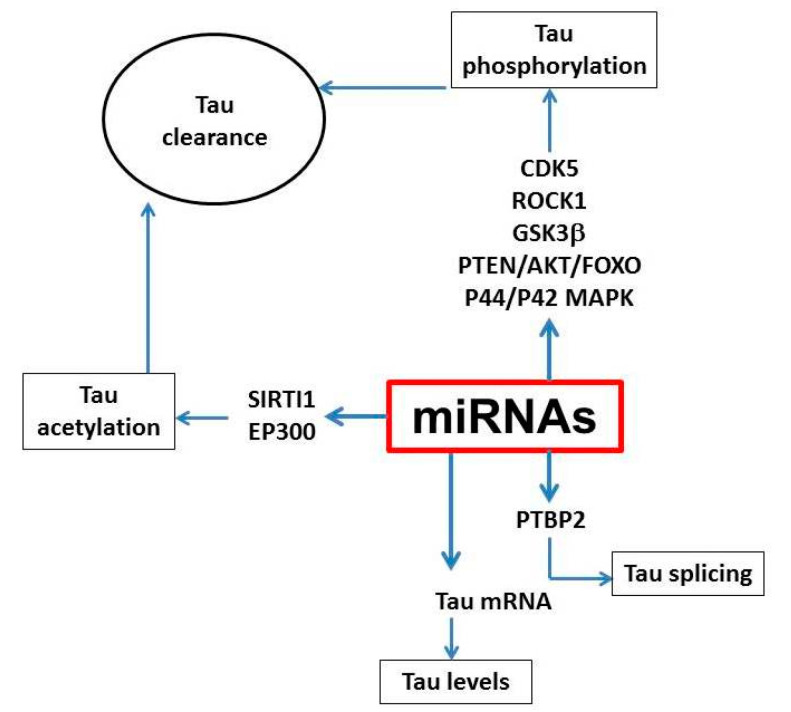
Tau metabolic pathways and molecular mechanisms targeted by miRNAs. Different miRNAs can target kinases and phosphatases, which regulate tau phosphorylation and acetylation whereby they can impair tau degradation and clearance. In addition, miRNAs can influence transcription of the tau gene and exons splicing.

From these initial studies, miRNAs have been studied in an effort to understand their biological link with tau pathology. To this end, many of them have been associated with neuroinflammation, apoptosis, neurogenesis and synaptic plasticity but only a few have been shown to participate directly in tau metabolism (Figure 1). Among them, miR-125, which is up-regulated in AD, promotes tau hyper-phosphorylation in neuronal cells via activation of the CDK5/P35 and p44/42-MAPK kinases, most likely through down-regulation of its target genes: the two phosphatases DUSP6 and PPP1CA. In support of this finding, direct hippocampal delivery of miR-125b mimic improved learning and memory, inhibited the expression of DUSP6, and PPP1CA, and significantly reduced aberrant tau phosphorylation in C57BL/6 mice [58]. Although mostly known for its key role in the inflammatory response, miR-146a has been recently shown to affect tau phosphorylation. Interestingly, miR-146a suppresses ROCK1, a kinase important for phosphorylation and activation of the phosphatase PTEN [59], thus, indirectly promoting tau pathology. Furthermore, miR-146a inhibition in 5xFAD mice successfully reduced hyper-phosphorylated tau and rescued memory dysfunction (Figure 1) [60].

Additionally, miR-138, another miRNA reported up-regulated in AD, was found to induce tau phosphorylation via the down-regulation of the RARA/GSK3β pathway in multiple in vitro models [60]. A very well characterized miRNA cluster is the miR-132/212. These miRNAs are down-regulated in tauopathy patients and transgenic models of the disease and have been linked to tau editing and synaptic function [61]. One of the first studies evaluating the role played by miR-132/212 in AD pathology was performed in the triple transgenic model of AD, the 3xTg mice. Notably, tau was identified as one of miR-132 direct target. In fact, loss of miR-132 increases levels of total and phosphorylated tau and promoted tau aggregation in these mice. Consistently, restoring miR-132 to normal levels improved tau pathology and long-term memory in these mice [61]. A follow-up study uncovered an additional mechanism underlying the exacerbation of both tau and Aβ pathology following down regulation of miR-132 levels in the hippocampus of APP/PS1 mice, involving Inositol trisphosphate 3-kinase B (ITPKB) kinase [62]. This kinase, which is up-regulated in AD, can activate BACE1 leading to Aβ production, but also promote tau phosphorylation via activation of ERK1/2 [63]. Furthermore, miR-132 can modulate neuronal survival by controlling PTEN/AKT/FOXO3 signaling as well as other genes (i.e., Rbfox1, GSK3β, EP300) [64,65] (Figure 1).

Beside phosphorylation, a few miRNAs have also been investigated for their possible contribution to tau clearance. It is known that some of the post-translational modifications of tau result in an impairment of its clearance via inhibition of ubiquitin binding thus promoting tau aggregation and mis-sorting. Reports suggest that SIRT1 gene is directly inhibited by miR-9, miR-132/212, and miR-181c [66]. SIRT1 deficiency enhanced levels of acetylated-tau and toxic forms of phosphorylated tau in primary neurons and in vivo [67] suggesting that dysregulation of these miRNAs that target this enzyme could significantly influence tau pathology by modulating tau clearance (Figure 1). In recent years, accumulating evidence suggests that microRNAs play an important role for the regulation of autophagy, another important tau clearance pathway. Thus, in vivo and in vitro evidence show that miR-34 by targeting Bcl-2 inhibits autophagy [68]. Others like miR-214-3p and miR299-5p, which are down-regulated in animal models of AD, can also negatively regulate autophagy by targeting autophagy related protein 5 (Atg5) [69]. In a similar fashion, miR-132/212, by targeting important autophagy proteins, such as Atg9a and Atg5-12, modulates autophagic flux in vivo [61].

Another important aspect of the neurobiology and metabolism of tau protein, which has been implicated also in the pathogenesis of tau neuropathology, is the editing process at the gene level. To this end, different miRNAs have been shown to modulate alternative splicing of the tau gene. Among them, miR-124, miR-9, miR 132, miR-137, and miR-153, have all been implicated for their capacity to control the alternative spicing of tau exon 10 in neuronal cells, by targeting splicing factors, such as poly-pyrimidine tract-binding protein 1 and 2 (PTBP1/2) [70]. Thus, one study reported that, compared with controls, post-mortem brain tissues of patients with PSP, a primary tauopathy with 4R repeats, have a significant down-regulation of miR-132 and up-regulation of PTPB2, while brains from Dicer KO mice were shown to have increased PTBP1 protein levels [48].

Overall the data presented so far show that miRNAs can contribute to the onset and progression of tau pathology by influencing kinases and phosphatases and splicing factors responsible for tau metabolism, as well tau clearing pathways. However, we still do not know whether miRNAs alterations in tauopathies are secondary to the disease process or rather upstream events responsible for the generation of post-translational modified tau isoforms, tau pro-aggregatory conformational changes, tau insoluble fibrils, and ultimately the progressive accumulation of all of these pathogenic forms. To start addressing this question, we recently performed a study in a tau transgenic mouse model, the hTau, which better recapitulates the human tau pathology, and for this reason considered as a relevant model of tauopathy. Thus, while most of the other models express mutant human tau transgenes, this model expresses of all six alternately spliced forms of normal human tau in absence of mouse tau. Importantly, the hTau mice display an age-related accumulation of phosphorylated tau, memory and cognitive impairment, together with reduced basal synaptic function [71].

To establish the miRNA expression profile in the brain of hTau mice in comparison with WT control, we performed an unbiased miRNA PCR array analysis in the cortex and hippocampus of 12-month-old mice, an age when tau neuropathology and cognitive impairments are well established [71,72]. The initial analysis revealed that hTau mice displayed many dysregulated miRNAs in these regions compared with WT. Among them, we selected the ones showing at least a two-fold change and were highly conserved between mouse and human (i.e., miR-132-3p, miR-146a-5p, miR-22-3p, and miR-455-5p). Validation by qRT-PCR analysis showed that at 12 months of age, miR-132-3p, miR-22-3p, miR-455-5p, and miR-146a-5p were significantly up-regulated in the hippocampus of hTau mice, but no changes were observed in the cortex. To assess whether these changes were present at earlier time points, we also measured their levels in the pre-symptomatic stage of the disease phenotype (three and six months of age). Although changes were modest, we found that at six months of age, miR-132-3p, miR-22-3p, and miR-146a-5p were up-regulated in the hippocampus of tau mice compared with WT controls. Notably, no changes were observed at three-months between the two groups [72]. Analyses of their predicted and validated target genes revealed that miR-22-3p targets SIRT1, p21, MeCP2, the brain-derived neurotrophic factor (BDNF), and PTEN (miRBase), a blocker of the AKT signaling pathway, which promotes tau phosphorylation and aggregation. On the other hand, miR-132-3p targets the 3′UTR of tau protein, the polypyrimidine tract binding protein 2 (PTBP2) [72,73], SIRT1, PTEN, MeCP2, and BDNF, whereas miR-146a suppresses ROCK kinase and induces tau phosphorylation via (Table 2). Enrichment analysis looking at the top signaling pathways modulated by these miRNAs showed: axonal guidance, neuregulin signaling for miR-132-3p; neuroinflammation and synaptogenesis signaling for miR146a-5p; and synaptogenesis and NGF signaling pathway for miR-22-3p. Interestingly, miR-132-3p and miR-22-3p shared 48 target genes, including PTEN, BDNF, MecP2 and SIRT1, all of which target mechanisms associated with tau pathology [72,73].

## 5. Conclusions and Future Directions

There is no doubt that, in recent years, we have made substantial progress in the understanding of this new class of non-coding RNA and their wide range of actions whereby they influence processes of functional importance for the pathogenesis of several neurodegenerative diseases including tauopathy. However, despite these advances, we are still in the early days and so there is a lot of work to be done in the effort to unravel the full neurobiology of miRNAs. In fact, while we know that many miRNA are dysregulated in tauopathies, what the molecular mechanisms are and to what degree specific miRNAs are directly or indirectly involved in the onset and progression of these diseases remain practically unknown.

Similar significant progress has also been made in the diverse technologies available for miRNA detection. In fact from the original real-time fluorescent quantitative PCR and digital PCR technology, in-situ hybridization, new microarray methods have been successfully established and widely implemented, while more recent advances include electrochemical detection based on enzymatic signal amplification, rolling ring amplification, and nanoparticle technology [73].

Thanks in part to such scientific and technological achievements some miRNAs have been already identified as candidate biomarkers which could potentially allow an earlier detection and possibly a subsequent higher rate of success for a treatment strategy. However, we still lack consistent and reproducible data on their expression in tissues such as CSF and blood, and the literature on this topic is inconsistent. It is possible that this fact is secondary to technical differences in the measurements and analysis as well as the heterogeneity of the samples that are collected (different stages of the diseases, co-existence of other clinical conditions). Therefore, there is an urgent need to conduct larger studies in patients with distinction to a precise stage of the disease, different brain areas, and specific cell types to successfully map miRNA changes in relation to the onset and progression of the neuropathology.

Finally, a comprehensive functional characterization of miRNAs in the context of the interaction among themselves (miRNA/miRNA), with other non-coding RNA species (i.e., circular RNAs, long non-coding RNAs), and with regular mRNAs leading to improved computational prediction algorithms of the biological events we discussed would be important not only for the field on miRNA research, but and most importantly to fully elucidate the causes/consequences relationship of their dysregulation in tauopathy.

In conclusion, while the literature supports that miRNAs play crucial functions in modulating several aspects of tau-related diseases, the exact role and implication of miRNA dysregulation in tauopathy pathogenesis remains to be fully elucidated. Moreover, whether a single miRNA or a combination of them (signature RNA) should be considered as the “ideal” therapeutic candidate for the treatment of these disorders requires further studies and more in depth analysis.

## Figures and Tables

**Table 1:** Major primary and secondary tauopathies.

**Primary Tauopathies**			
	**Tau Isoform**	**Neurons**	**Astrocytes**
*Progressive supranuclear palsy*	3R	NFTs	Tufted astrocytes
*Pick’s disease*	4R	Pick’s bodies	Tufted astrocytes
*Corticobasal degeneration*	4R	NFTs, neurophil threads	Astrocytic plaques
*Frontotemporal dementia with parkinsonism linked to chromosome 17*	3R, 4R, 3R+4R	NFTs	Yes
*Argyrophilic grain disease*	4R	Argyrophilic grains	No
*Globular glial tauopathy*	4R	NFTs, neurophil threads	Globular inclusions
**Secondary Tauopathies**			
*Alzheimer’s disease*	3R, 4R	NFTs, tau oligomers	No
*Down syndrome*	3R, 4R	NFTs	No

3R; 3repeats; 4R: 4 repeats; NFTs: neurofibrillary tangles.

**Table 2:** Major predicted and validated targets gens for miRNAs that are dysregulated in the hippocampi of hTau mice compared with matched wild type controls at an early stage of their phenotype (6 months).

miRNA	Target Gene	Effect on Tau
miRNA 22-3p	SIRTI 1	Tau phosphorylation
	P21	Tau acetylation
	MeCP2	
	PTEN	
miRNA132-3p	PTBP2	Tau phosphorylation
	SIRTI 1	Tau acetylation
	PTEN	Tau splicing
	MeCP2	
miRNA146a-5p	ROCK1	Tau phosphorylation

## References

[1] Jadhav S., Avila J., Schöll M., Kovacs G.G., Kövari E., Skrabana R., Evans L.D., Kontsekova E., Malawska B., de Silva R. (2019). A walk through tau therapeutic strategies. Acta Neuropathol. Commun..

[2] Lauretti E., Praticò D. (2020). Alzheimer’s disease: Phenotypic approaches using disease models and the targeting of tau protein. Expert Opin. Ther. Targets.

[3] Anstey K.J., Cherbuin N., Herath P.M. (2013). Development of a new method for assessing global risk of Alzheimer’s disease for use in population health approaches to prevention. Prev. Sci..

[4] Ninomiya T., Ohara T., Hirakawa Y., Yoshida D., Doi Y., Hata J., Kanba S., Iwaki T., Kiyohara Y. (2011). Midlife and late-life blood pressure and dementia in Japanese elderly: The Hisayama study. Hypertension.

[5] Edwards G., Zhao J., Dash P.K., Soto C., Moreno-Gonzalez I. (2020). Traumatic brain injury induces tau aggregation and spreading. J. Neurotrauma.

[6] Zhang X., Gao F., Wang D., Li C., Fu Y., He W., Zhang J. (2018). Tau Pathology in Parkinson’s Disease. Front. Neurol..

[7] Moussaud S., Jones D.R., Moussaud-Lamodière E.L., Delenclos M., Ross O.A., McLean P.J. (2014). Alpha-synuclein and tau: Teammates in neurodegeneration?. Mol. Neurodegener..

[8] Iqbal K., Liu F., Gong C.X., Grundke-Iqbal I. (2010). Tau in Alzheimer disease and related tauopathies. Curr. Alzheimer Res..

[9] Fitzpatrick A.W.P., Falcon B., He S., Murzin A.G., Murshudov G., Garringer H.J., Growther R.A., Ghetti B., Goedert M., Scheres S.H.W. (2017). Cryo-EM structures of tau filaments from Alzheimer’s disease. Nature.

[10] Li C., Götz J. (2017). Tau-based therapies in neurodegeneration: Opportunities and challenges. Nat. Rev. Drug Discov..

[11] Irwin D.J. (2016). Tauopathies as clinicopathological entities. Parkinsonism Relat. Disord..

[12] Braak H., Alafuzoff I., Arzberger T., Kretzschmar H., Del Tredici K. (2006). Staging of Alzheimer’s disease-associated neurofibrillary pathology using paraffin sections and immunocytochemistry. Acta Neuropathol..

[13] Wolfe M.S. (2009). Tau mutations in neurodegenerative diseases. J. Biol. Chem..

[14] Huang Y., Zhang J.L., Yu X.L., Xu T.S., Wang Z.B., Cheng X.C. (2013). Molecular functions of small regulatory noncoding RNA. Biochemistry.

[15] Morris K.V., Mattick J.S. (2014). The rise of regulatory RNA. Nat. Rev. Genet..

[16] David P.B. (2018). Metazoan MicroRNAs. Cell.

[17] Sempere L.F., Freemantle S., Pitha-Rowe I., Moss E., Dmitrovsky E., Ambros V. (2004). Expression profiling of mammalian microRNAs uncovers a subset of brain-expressed microRNAs with possible roles in murine and human neuronal differentiation. Genome Biol..

[18] Landgraf P., Rusu M., Sheridan R., Sewer A., Iovino N., Aravin A., Pfeffer S., Rice A., Kamphorst A., Landthaler M. (2007). A mammalian microRNA expression atlas based on small RNA library sequencing. Cell.

[19] Bak M., Silahtaroglu A., Møller M., Christensen M., Rath M.F., Skryabin B., Tommerup N., Kauppinen S. (2008). MicroRNA expression in the adult mouse central nervous system. RNA.

[20] Lugli G., Torvik V.I., Larson J., Smalheiser N.R. (2008). Expression of microRNAs and their precursors in synaptic fractions of adult mouse forebrain. J. Neurochem..

[21] Natera-Naranjo O., Aschrafi A., Gioio A.E., Kaplan B.B. (2010). Identification and quantitative analyses of microRNAs located in the distal axons of sympathetic neurons. RNA.

[22] Makeyev E.V., Zhang J., Carrasco M.A., Maniatis T. (2007). The MicroRNA miR-124 promotes neuronal differentiation by triggering brain-specific alternative pre-mRNA splicing. Mol. Cell.

[23] He Y., Li H.B., Li X., Zhou Y., Xia X.B., Song W.T. (2018). MiR-124 promotes the growth of retinal ganglion cells derived from Müller cells. Cell Physiol. Biochem..

[24] Radhakrishnan B., Alwin Prem Anand A. (2016). Role of miRNA-9 in brain development. J. Exp. Neurosci..

[25] Dajas-Bailador F., Bonev B., Garcez P., Stanley P., Guillemot F., Papalopulu N. (2012). microRNA-9 regulates axon extension and branching by targeting Map1b in mouse cortical neurons. Nat. Neurosci..

[26] Otaegi G., Pollock A., Hong J., Sun T. (2011). MicroRNA miR-9 modifies motor neuron columns by a tuning regulation of FoxP1 levels in developing spinal cords. J. Neurosci..

[27] Konopka W., Kiryk A., Novak M., Herwerth M., Parkitna J.R., Wawrzyniak M., Kowarsch A., Michaluk P., Dzwonek J., Arnsperger T. (2010). MicroRNA loss enhances learning and memory in mice. J. Neurosci..

[28] Huang Y.-W., Ruiz C.R., Eyler E.C., Lin K., Meffert M.K. (2012). Dual regulation of miRNA biogenesis generates target specificity in neurotrophin-induced protein synthesis. Cell.

[29] Weiss K., Antoniou A., Schratt G. (2015). Non-coding mechanisms of local mRNA translation in neuronal dendrites. Eur. J. Cell Biol..

[30] Guo C.H., Cao T., Zheng L.T., Waddington J.L., Zhen X.C. (2020). Development and characterization of an inducible Dicer conditional knockout mouse model of Parkinson’s disease: Validation of the antiparkinsonian effects of a sigma-1 receptor agonist and dihydromyricetin. Acta Pharmacol. Sin..

[31] Emde A., Eitan C., Liou L.L., Libby R.T., Rivkin N., Magen I., Reichenstein I., Oppenheim H., Eilam R., Silvestroni A. (2015). Dysregulated miRNA biogenesis downstream of cellular stress and ALS-causing mutations: A new mechanism for ALS. EMBO J..

[32] Cheng S., Zhang C., Xu C., Wang L., Zou X., Chen G. (2014). Age-dependent neuron loss is associated with impaired adult neurogenesis in forebrain neuron-specific Dicer conditional knockout mice. Int. J. Biochem. Cell Biol..

[33] Hansen K.F., Sakamoto K., Wayman G.A., Impey S., Obrietan K. (2010). Transgenic miR132 alters neuronal spine density and impairs novel object recognition memory. PLoS ONE.

[34] Edbauer D., Neilson J.R., Foster K.A., Wang C.F., Seeburg D.P., Batterton M.N., Tada T., Dolan B.M., Sharp P.A., Shen M. (2010). Regulation of synaptic structure and function by FMRP-associated microRNAs miR-125b and miR-132. Neuron.

[35] Lin Q., Wei W., Coelho C.M., Li X., Baker-Andresen D., Dudley K., Ratnu V.S., Boskovic Z., Kobor M.S., Sun Y.E. (2011). The brain-specific microRNA miR-128b regulates the formation of fear-extinction memory. Nat. Neurosci..

[36] Rajasethupathy P., Fiumara F., Sheridan R., Betel D., Puthanveettil S.V., Russo J.J., Sander C., Tuschl T., Kandel E. (2009). Characterization of small RNAs in Aplysia reveals a role for miR-124 in constraining synaptic plasticity through CREB. Neuron.

[37] Gao J., Wang W.Y., Mao Y.W., Gräff J., Guan J.S., Pan L., Mak G., Kim D., Su S.C., Tsai L.-H. (2010). A novel pathway regulates memory and plasticity via SIRT1 and miR-134. Nature.

[38] Taganov K.D., Boldin M.P., Chang K.J., Baltimore D. (2006). NF-kappaB-dependent induction of microRNA miR-146, an inhibitor targeted to signaling proteins of innate immune responses. Proc. Natl. Acad. Sci. USA.

[39] Su W., Aloi M.S., Garden G.A. (2016). MicroRNAs mediating CNS inflammation: Small regulators with powerful potential. Brain Behav. Immun..

[40] Cho K.H.T., Xu B., Blenkiron C., Fraser M. (2019). Emerging roles of miRNAs in brain development and perinatal brain injury. Front. Physiol..

[41] Sharma S., Lu H.C. (2018). microRNAs in Neurodegeneration: Current findings and potential impacts. J. Alzheimers Dis. Parkinsonism.

[42] Catanesi M., d’Angelo M., Tupone M.G., Benedetti E., Giordano A., Castelli V., Cimini A. (2020). MicroRNAs dysregulation and mitochondrial dysfunction in neurodegenerative diseases. Int. J. Mol. Sci..

[43] Wang M., Qin L., Tang B. (2019). MicroRNAs in Alzheimer’s disease. Front. Genet..

[44] Angelucci F., Cechova K., Valis M., Kuca K., Zhang B., Hort J. (2019). MicroRNAs in Alzheimer’s disease: Diagnostic markers or therapeutic agents?. Front. Pharmacol..

[45] Cosín-Tomás M., Antonell A., Lladó A., Alcolea D., Fortea J., Ezquerra M., Lleo A., Marti M.J., Pallas M., Sanchez-Valle R. (2017). Plasma miR-34a-5p and miR-545-3p as early biomarkers of Alzheimer’s disease: Potential and limitations. Mol. Neurobiol..

[46] Kumar S., Reddy P.H. (2018). MicroRNA-455-3p as a Potential biomarker for Alzheimer’s disease: An update. Front. Aging Neurosci..

[47] Kiko T., Nakagawa K., Tsuduki T., Furukawa K., Arai H., Miyazawa T. (2014). MicroRNAs in plasma and cerebrospinal fluid as potential markers for Alzheimer’s disease. J. Alzheimer’s Dis..

[48] Smith P.Y., Delay C., Girard J., Papon M.A., Planel E., Sergeant N. (2011). MicroRNA-132 loss is associated with tau exon 10 inclusion in progressive supranuclear palsy. Hum. Mol. Genet..

[49] Tatura R., Buchholz M., Dickson D.W., van Swieten J., McLean C., Höglinger G., Muller U. (2016). microRNA profiling: Increased expression of miR-147a and miR-518e in progressive supranuclear palsy (PSP). Neurogenetics.

[50] Starhof C., Hejl A.M., Heegaard N.H.H., Carlsen A.L., Burton M., Lilje B., Winge K. (2019). The biomarker potential of cell-free microRNA from cerebrospinal fluid in Parkinsonian syndromes. Mov. Disord..

[51] Maldonado-Lasuncion I., Atienza M., Sanchez-Espinosa M.P., Cantero J.L. (2019). Aging-related changes in cognition and cortical integrity are associated with serum expression of candidate microRNAs for Alzheimer’s disease. Cereb. Cortex.

[52] Geekiyanage H., Jicha G.A., Nelson P.T., Chan C. (2012). Blood serum miRNA: Non-invasive biomarkers for Alzheimer’s disease. Exp. Neurol..

[53] Schonrock N., Humphreys D.T., Preiss T., Götz J. (2012). Target gene repression mediated by miRNAs miR-181c and miR-9 both of which are down-regulated by amyloid-β. J. Mol. Neurosci..

[54] Galimberti D., Villa C., Fenoglio C., Serpente M., Ghezzi L., Cioffi S.M., Andrea A., Giorgio F., Elio S. (2014). Circulating miRNAs as potential biomarkers in Alzheimer’s disease. J. Alzheimer’s Dis..

[55] Backes C., Meese E., Keller A. (2016). Specific miRNA disease biomarkers in blood, serum and plasma: Challenges and prospects. Mol. Diagn. Ther..

[56] Hébert S.S., Papadopoulou A.S., Smith P., Galas M.C., Planel E., Silahtaroglu A.N., Sergeant N., Buee L., De Stropper B. (2010). Genetic ablation of Dicer in adult forebrain neurons results in abnormal tau hyperphosphorylation and neurodegeneration. Hum. Mol. Genet..

[57] Bilen J., Liu N., Burnett B.G., Pittman R.N., Bonini N.M. (2006). MicroRNA pathways modulate polyglutamine-induced neurodegeneration. Mol. Cell.

[58] Banzhaf-Strathmann J., Benito E., May S., Arzberger T., Tahirovic S., Kretzschmar H., Fischer A., Edbauer D. (2014). MicroRNA-125b induces tau hyperphosphorylation and cognitive deficits in Alzheimer’s disease. EMBO J..

[59] Vemula S., Shi J., Hanneman P., Wei L., Kapur R. (2010). ROCK1 functions as a suppressor of inflammatory cell migration by regulating PTEN phosphorylation and stability. Blood.

[60] Wang G., Huang Y., Wang L.L., Zhang Y.F., Xu J., Zhou Y., Lourenco G.F., Zhang B., Wang Y., Ren R.J. (2016). MicroRNA-146a suppresses ROCK1 allowing hyperphosphorylation of tau in Alzheimer’s disease. Sci. Rep..

[61] Smith P.Y., Hernandez-Rapp J., Jolivette F., Lecours C., Bisht K., Goupil C. (2015). miR-132/212 deficiency impairs tau metabolism and promotes pathological aggregation in vivo. Hum. Mol. Genet..

[62] Salta E., Sierksma A., Vanden Eynden E., De Strooper B. (2016). miR-132 loss de-represses ITPKB and aggravates amyloid and tau pathology in Alzheimer’s brain. EMBO Mol. Med..

[63] Stygelbout V., Leroy K., Pouillon V., Ando K., D’Amico E., Jia Y. (2014). Inositol trisphosphate 3-kinase B is increased in human Alzheimer brain and exacerbates mouse Alzheimer pathology. Brain.

[64] Wong H.K., Veremeyko T., Patel N., Lemere C.A., Walsh D.M., Esau C., Vanderburg C., Krichevsky A.M. (2013). De-repression of FOXO3a death axis by microRNA-132 and -212 causes neuronal apoptosis in Alzheimer’s disease. Hum. Mol. Genet..

[65] El Fatimy R., Li S., Chen Z., Mushannen T., Gongala S., Wei Z., Balu D.T., Rabinovsky R., Adam Cantlon A., Elkhal A. (2018). MicroRNA-132 provides neuroprotection for tauopathies via multiple signaling pathways. Acta Neuropathol..

[66] Zhao J., Yue D., Zhou Y., Jia L., Wang H., Guo M., Xu H., Chen C., Zhang J., Xu L. (2017). The role of microRNAs in Aβ deposition and tau phosphorylation in Alzheimer’s disease. Front. Neurol..

[67] Min S.W., Cho S.H., Zhou Y., Schroeder S., Haroutunian V., Seeley W.W., Huang E.J., Shen Y., Masliah E., Mukherjee C. (2010). Acetylation of tau inhibits its degradation and contributes to tauopathy. Neuron.

[68] Zhang Y., Li Q., Liu C., Gao S., Ping H., Wang J., Wang P. (2016). MiR-214-3p attenuates cognition defects via the inhibition of autophagy in SAMP8 mouse model of sporadic Alzheimer’s disease. Neurotoxicology.

[69] Zhang Y., Liu C., Wang J., Li Q., Ping H., Gao S., Wang P. (2016). MiR-299-5p regulates apoptosis through autophagy in neurons and ameliorates cognitive capacity in APPswe/PS1dE9 mice. Sci. Rep..

[70] Bazrgar M., Khodabakhsh P., Mohagheghi F., Prudencio M., Ahmadiani A. (2020). Brain microRNAs dysregulation: Implication for mis-splicing and abnormal post-translational modifications of tau protein in Alzheimer’s disease and related tauopathies. Pharmacol. Res..

[71] Polydoro M., Acker C.M., Duff K., Castillo P.E., Davies P. (2009). Age-dependent impairment of cognitive and synaptic function in the htau mouse model of tau pathology. J. Neurosci..

[72] Lauretti E., Dincer O., Praticò D. (2020). Regional and temporal miRNAs expression profile in a transgenic mouse model of tauopathy: Implication for its pathogenesis. Mol. Psychiatry.

[73] Liu K., Tong H., Li T., Wang X., Chen Y. (2020). Research progress in molecular biology related quantitative methods of MicroRNA. Am. J. Transl. Res..

